# Digestive Diseases and Frailty in Middle-Aged and Older Chinese Adults: A Longitudinal Study

**DOI:** 10.1093/geroni/igaf122.2367

**Published:** 2025-12-31

**Authors:** Hongmei Jiao, Jiaxin Li

**Affiliations:** Peking University First Hospital, Beijing, Beijing, China (People’s Republic); Peking University First Hospital, Beijing, Beijing, China (People’s Republic)

## Abstract

Frailty, characterized by reduced physiological reserves and diminished stress response, is linked to chronic diseases. However, its association with nonneoplastic digestive diseases remains underexplored. This study investigates this relationship among middle-aged and older Chinese adults using data from the 2011–2018 China Health and Retirement Longitudinal Study (CHARLS). Frailty was assessed using a 32-item frailty index (FI > 0.25), and nonneoplastic digestive diseases were identified through self-reported diagnoses. Cox proportional hazards models and Restricted Cubic Splines (RCS) were employed to examine associations, while mediation analysis evaluated the roles of inflammation-related markers (leukocytes, platelets, platelet-to-leukocyte ratio [PWR], and C-reactive protein [CRP]) and nutrition-related markers (triglycerides [TG] and glycosylated hemoglobin [HbA1c]). Among 8,507 non-frail participants (mean age 59.9 ± 9.7 years, 56.4% female), 24.0% reported nonneoplastic digestive diseases. Over a 7-year follow-up, 2,397 frailty cases were documented. Nonneoplastic digestive diseases independently increased frailty risk (HR = 1.250, 95% CI: 1.100–1.430, P < 0.001). RCS revealed a non-linear relationship. Mediation analysis indicated that TG and HbA1c mediated -0.998% and -2.26% of the association, respectively, while inflammation-related markers showed no significant mediation. These findings underscore the link between nonneoplastic digestive diseases and frailty, highlighting the importance of early intervention and nutritional strategies to mitigate frailty risk in older persons.

